# Origin of B-Cell Neoplasms in Autoimmune Disease

**DOI:** 10.1371/journal.pone.0158360

**Published:** 2016-06-29

**Authors:** Kari Hemminki, Xiangdong Liu, Jianguang Ji, Asta Försti

**Affiliations:** 1 Division of Molecular Genetic Epidemiology, German Cancer Research Center (DKFZ), Im Neuenheimer Feld 580, D-69120, Heidelberg, Germany; 2 Center for Primary Health Care Research, Lund University, 205 02, Malmö, Sweden; 3 Department of Measles, National Institute for Viral Disease Control and Prevention, Chinese Center for Disease Control and Prevention, Beijing, China; IRCCS National Cancer Institute, ITALY

## Abstract

Autoimmune diseases (ADs) are associated with a number of B-cell neoplasms but the associations are selective in regard to the type of neoplasm and the conferred risks are variable. So far no mechanistic bases for these differential associations have been demonstrated. We speculate that developmental origin of B-cells might propose a mechanistic rationale for their carcinogenic response to autoimmune stimuli and tested the hypothesis on our previous studies on the risks of B-cell neoplasms after any of 33 ADs. We found that predominantly germinal center (GC)-derived B-cells showed multiple associations with ADs: diffuse large B cell lymphoma associated with 15 ADs, follicular lymphoma with 7 ADs and Hodgkin lymphoma with 11 ADs. Notably, these neoplasms shared significant associations with 5 ADs (immune thrombocytopenic purpura, polymyositis/dermatomyositis, rheumatoid arthritis, Sjogren syndrome and systemic lupus erythematosis). By contrast, primarily non-GC neoplasms, acute lymphocytic leukemia, chronic lymphocytic leukemia and myeloma associated with 2 ADs only and mantle cell lymphoma with 1 AD. None of the neoplasms shared associated ADs. These data may suggest that autoimmune stimulation critically interferes with the rapid cell division, somatic hypermutation, class switch recombination and immunological selection of maturing B-cell in the GC and delivers damage contributing to transformation.

## Introduction

Autoimmune diseases (ADs) are characterized by the reaction of T or B lymphocytes towards own (self or auto) antigens and destruction of body’s own constituents [1, 2]. ADs are a heterogeneous group of diseases, either with a systemic or a localized presentation and with or without circulating autoantibodies. In Caucasian populations, the prevalence of individual ADs varies from very rare to moderately common, however, jointly reaching a population prevalence of up to 10% [3]. With the exception of some rare Mendelian diseases, most ADs are considered to be multifactorial diseases with a complex environmental and genetic background [4]. In addition to the strong contribution by the HLA locus, numerous susceptibility genes and loci are known, many shared by several ADs and some acting even antagonistically [4, 5]. ADs are associated with a number of cancers, including B-cell neoplasms [6]. However, the diverse ADs appear to be associated with diverse neoplasms and no mechanistic bases for the selective response have been demonstrated. In a recent study, ADs were divided into those where the autoimmune activation was thought to be through B- or T-cells but no clear pattern could be seen on B- or T-cell lymphomas [7].

We speculated that developmental origin of B-cells might propose a mechanistic rationale for their carcinogenic response to autoimmune stimuli. It has been suggested that most B-cell lymphomas derive from germinal center (GC) cells which are undergoing somatic hypermutation and class switch recombination [8]. We hypothesized accordingly that B-cell neoplasms originating from GC cells are most responsive to autoimmune stimulation and that these neoplasms show patterns of similarity in their response to different ADs. We tested the hypothesis on a series of studies in which we analyzed the risk of B-cell neoplasms after any of 33 ADs in Sweden [9–12].

## Methods

We adopted the cell of origin classification of Seifert and coworkers [8]. As GC-dereived cell types were considered diffuse large B-cell lymphoma (DLBCL, of which the majority is CG-derived and the minority is post-GC cells), follicular lymphoma, Hodgkin lymphoma (of which the predominant proportion is classical Hodgkin lymphoma, originating from pre-apoptotic GC B-cells). Pre-GC-derived tumors were acute lymphocytic leukemia (ALL) and non-mutated chronic lymphocytic leukemia (CLL, >60% pre GC while a minority is post-GC-tumors with IGH mutations). Multiple myeloma was the only pure post-GC type while mantle cell lymphoma is assumed to be derived from CD5+ mantle zone cells.

AD patients were identified from 4 published studies based on the Swedish Hospital Discharge Register operating regionally since 1964 and nation-wide since 1986 [9–12]. The studies on myeloma and leukemia were followed up to 2008 and 402,462 AD patients were identified. For the studies on lymphoma, the Outpatient Registry (2001–2010), and Primary Health Care Registry in Stockholm and Region Skane (2001–2010) were additionally included and 878,161 AD patients were identified. Cancer cases diagnosed after AD were obtained from the nation-wide Swedish Cancer Registry. The original studies included 3096 non-Hodgkin lymphoma (NHL) patients (however, a smaller number of specific NHL subtypes were available because the codes were used from 1993 onwards), 371 Hodgkin lymphoma patients, 1128 leukemia patients and 457 myeloma patients [9–12]. We included in the present study specific B-cell types on which we had data on at least 50 cases with combined ADs.

Person-years of follow-up were calculated from date of discharge with the first main diagnosis of AD until death, emigration, or closing date, December 31, 2008 or 2010. Standardized incidence ratios (SIRs) were calculated as the ratio of observed (O) to expected number of cases. Expected numbers were calculated for anyone not hospitalized for AD. The expected numbers were calculated as age, sex, period, region and socioeconomic status-specific standardized incidence rates. Additional adjustments were made for hospitalization for obesity, chronic obstructive pulmonary disease (surrogate of smoking) and alcohol use disorders. We show data for ADs with at least 3 neoplasms in the most common AD category. Differences were called ‘significant’ when the 95% confidence intervals (95%CIs) did not overlap with 1.00.

The study was approved by the regional ethical review board at Lund. The methods were carried out in accordance with the approved guidelines; only anonymized data and no human tissues were used.

## Results

**Fig 1:**
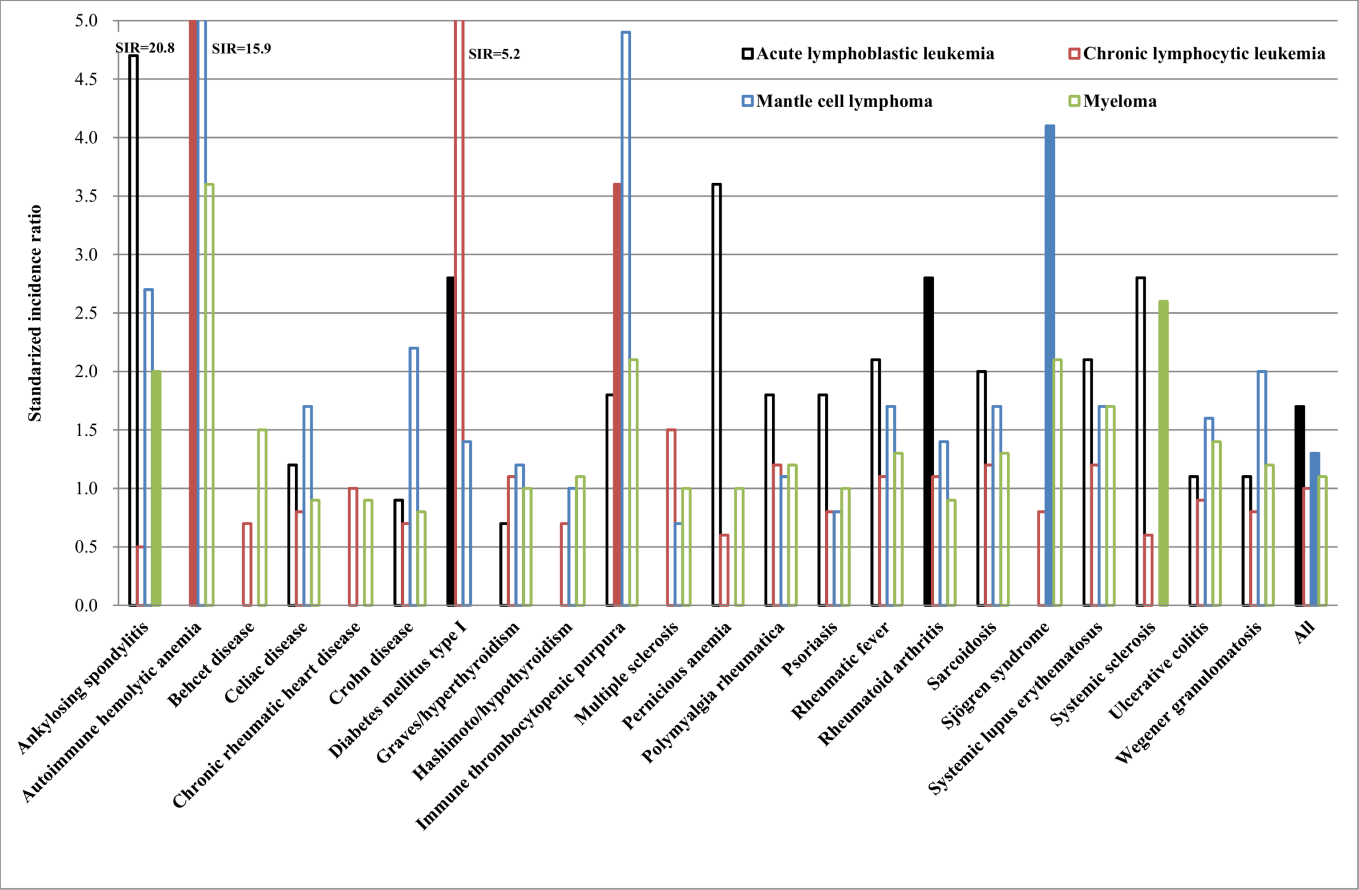
SIR for non-germinal center neoplasms in autoimmune disease patients. Red bars show significant and blue bars not significant associations.

Fig 1 shows results for non-GC-B-cell diseases. Significant associations are shown in red bars. ALL, CLL and myeloma associated with 2 ADs and mantle cell lymphoma only with 1 AD. Notably, none of the neoplasms shared associated ADs. The association of CLL with autoimmune hemolytic anemia (20.8) was high. Case numbers, SIRs and 95%CI are shown in Table 1.

**Table 1:** SIRs for non-germinal center neoplasms in autoimmune disease patients.

Autoimmune disease	Acute lymphoblastic leukemia				Chronic lymphocytic leukemia				Mantle cell lymphoma				Myeloma			
	O	SIR	95%CI		O	SIR	95%CI		O	SIR	95%CI		O	SIR	95%CI	
Ankylosing spondylitis	3	4.7	0.9	14.0	3	0.5	0.1	1.4	4	2.7	0.7	7.0	16	**2.0**	1.2	3.3
Autoimmune hemolytic anemia	0				12	**20.8**	10.7	36.4	1	15.9	0.0	91.2	3	3.6	0.7	10.5
Behcet disease	0				2	0.7	0.1	2.4	0				6	1.5	0.5	3.2
Celiac disease	5	1.2	0.4	2.9	2	0.8	0.1	2.9	2	1.7	0.2	6.3	3	0.9	0.2	2.5
Chronic rheumatic heart disease	0				17	1	0.6	1.6	0				23	0.9	0.6	1.4
Crohn disease	2	0.9	0.1	3.5	9	0.7	0.3	1.3	6	2.2	0.8	4.7	15	0.8	0.5	1.4
Diabetes mellitus type I	10	**2.8**	1.3	5.1	3	5.2	1.0	15.5	16	1.4	0.8	2.3	0			
Graves/hyperthyroidism	2	0.7	0.1	2.4	37	1.1	0.8	1.6	6	1.2	0.4	2.5	52	1.0	0.8	1.4
Hashimoto/hypothyroidism	0				6	0.7	0.3	1.5	4	1.0	0.3	2.4	15	1.1	0.6	1.9
Immune thrombocytopenic purpura	1	1.8	0.0	10.5	5	**3.6**	1.1	8.4	2	4.9	0.5	17.9	4	2.1	0.6	5.5
Multiple sclerosis	0				14	1.5	0.8	2.6	1	0.7	0.0	4.0	13	1.0	0.5	1.7
Pernicious anemia	2	3.6	0.3	13.2	5	0.6	0.2	1.3	0				13	1.0	0.5	1.7
Polymyalgia rheumatica	3	1.8	0.3	5.3	31	1.2	0.8	1.7	6	1.1	0.4	2.3	43	1.2	0.9	1.6
Psoriasis	3	1.8	0.4	5.5	13	0.8	0.4	1.3	7	0.8	0.3	1.8	23	1.0	0.6	1.5
Rheumatic fever	1	2.1	0.0	11.9	5	1.1	0.4	2.6	1	1.7	0.0	10.0	8	1.3	0.6	2.7
Rheumatoid arthritis	14	**2.8**	1.5	4.7	67	1.1	0.8	1.4	13	1.4	0.8	2.5	81	0.9	0.7	1.1
Sarcoidosis	2	2.0	0.2	7.3	12	1.2	0.6	2.0	3	1.7	0.3	5.1	19	1.3	0.8	2.0
Sjögren syndrome	0				1	0.8	0.0	4.5	4	**4.1**	1.1	10.6	4	2.1	0.6	5.4
Systemic lupus erythematosus	1	2.1	0.0	12.1	5	1.2	0.4	2.8	1	1.7	0.0	9.8	11	1.7	0.9	3.1
Systemic sclerosis	2	2.8	0.3	10.3	3	0.6	0.1	1.7	0				19	**2.6**	1.6	4.1
Ulcerative colitis	3	1.1	0.2	3.2	18	0.9	0.5	1.4	8	1.6	0.7	3.2	38	1.4	1.0	1.9
Wegener granulomatosis	1	1.1	0.0	6.4	11	0.8	0.4	1.4	2	2.0	0.2	7.4	26	1.2	0.8	1.7
All	58	**1.7**	1.3	2.2	291	1.0	0.9	1.2	81	**1.3**	1.1	1.7	457	1.1	1.0	1.2

Bold type indicates that the 95% CI does not include 1.00. Abbreviations: O, observed; SIR, standardized incidence ratio; CI, confidence interval.

**Fig 2:**
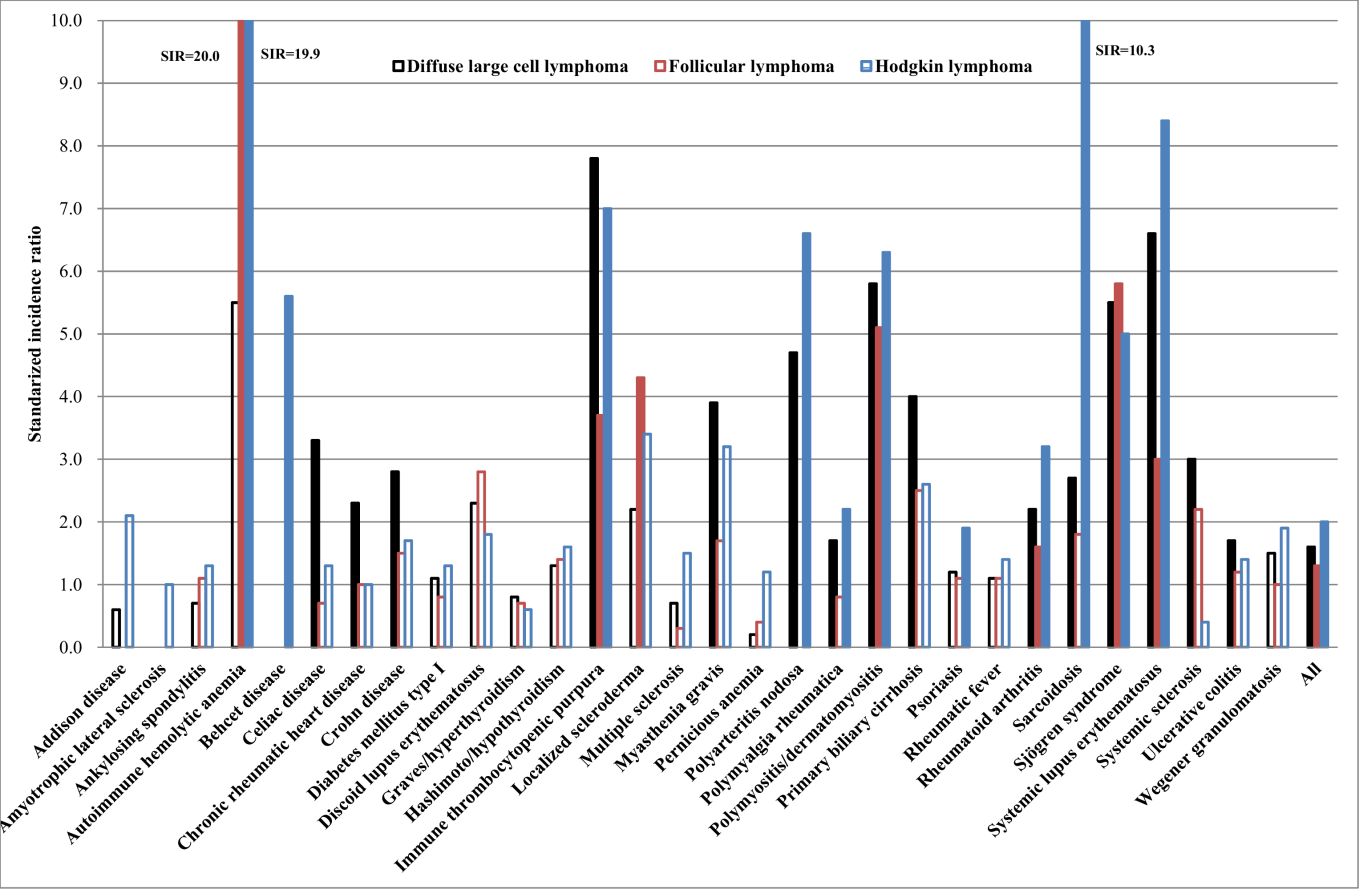
SIRs for primarily germinal center-derived neoplasms in autoimmune disease patients. Red bars show significant and blue bars not significant associations.

In Fig 2 we collected data on GC-derived neoplasms. Again case numbers, SIRs and 95%CI are shown in Table 2. Hodgkin data include all histological types, as these were predominantly GC-derived. DLBCL associated with 15 ADs, follicular lymphoma with 7 ADs and Hodgkin lymphoma with 11 ADs. Notably, these neoplasms shared significant associations with 5 ADs (immune thrombocytopenic purpura, polymyositis/dermatomyositis, rheumatoid arthritis, Sjogren syndrome and systemic lupus erythematosis). Moreover, the SIRs were almost equal for polymyositis/dermatomyositis and Sjogren syndrome between the three neoplasms. Another 4 AD associations were shared with the 2 neoplasms, and for follicular lymphoma (SIR 20.0) and Hodgkin lymphomas (19.9) the associations with autoimmune hemolytic anemia were excessive and equally high.

**Table 2:** SIRs for primarily germinal center-derived neoplasms in autoimmune disease patients.

Autoimmune disease	Diffuse large cell lymphoma				Follicular lymphoma				Hodgkin lymphoma			
	O	SIR	95%CI		O	SIR	95%CI		O	SIR	95%CI	
Addison disease	1	0.6	0.0	3.6	0				2	2.1	0.2	7.6
Amyotrophic lateral sclerosis	0				0				1	1.0	0.0	5.4
Ankylosing spondylitis	5	0.7	0.2	1.5	5	1.1	0.3	2.5	5	1.3	0.4	3.0
Autoimmune hemolytic anemia	2	5.5	0.5	20.4	5	**20.0**	6.3	46.9	6	**19.9**	7.2	43.6
Behcet disease	0				0				10	**5.6**	2.7	10.3
Celiac disease	25	**3.3**	2.1	4.9	3	0.7	0.1	2.0	9	1.3	0.6	2.4
Chronic rheumatic heart disease	22	**2.3**	1.4	3.5	6	1.0	0.4	2.2	7	1.0	0.4	2.1
Crohn disease	46	**2.8**	2.1	3.7	16	1.5	0.8	2.4	18	1.7	1.0	2.7
Diabetes mellitus type I	70	1.1	0.9	1.4	25	0.8	0.5	1.2	26	1.3	0.8	1.9
Discoid lupus erythematosus	4	2.3	0.6	6.0	3	2.8	0.5	8.2	1	1.8	0.0	10.0
Graves/hyperthyroidism	32	0.8	0.6	1.2	19	0.7	0.4	1.1	11	0.6	0.3	1.1
Hashimoto/hypothyroidism	40	1.3	0.9	1.7	28	1.4	0.9	2.0	16	1.6	0.9	2.6
Immune thrombocytopenic purpura	19	**7.8**	4.7	12.1	5	**3.7**	1.2	8.6	9	**7.0**	3.2	13.3
Localized scleroderma	3	2.2	0.4	6.5	4	**4.3**	1.1	11.2	2	3.4	0.3	12.3
Multiple sclerosis	6	0.7	0.2	1.4	2	0.3	0.0	1.1	8	1.5	0.6	2.9
Myasthenia gravis	8	**3.9**	1.7	7.8	2	1.7	0.2	6.1	3	3.2	0.6	9.5
Pernicious anemia	1	0.2	0.0	1.4	1	0.4	0.0	2.1	5	1.2	0.4	2.7
Polyarteritis nodosa	4	**4.7**	1.2	12.1	0				3	**6.6**	1.2	19.5
Polymyalgia rheumatica	57	**1.7**	1.3	2.2	16	0.8	0.5	1.4	20	**2.2**	1.4	3.5
Polymyositis/dermatomyositis	9	**5.8**	2.6	11.0	5	**5.1**	1.6	12.0	5	**6.3**	2.0	14.9
Primary biliary cirrhosis	7	**4.0**	1.6	8.2	3	2.5	0.5	7.4	1	2.6	0.0	14.7
Psoriasis	57	1.2	0.9	1.5	31	1.1	0.7	1.5	35	**1.9**	1.3	2.6
Rheumatic fever	3	1.1	0.2	3.2	2	1.1	0.1	4.1	3	1.4	0.3	4.2
Rheumatoid arthritis	126	**2.2**	1.8	2.6	57	**1.6**	1.2	2.0	93	**3.2**	2.6	3.9
Sarcoidosis	27	**2.7**	1.8	3.9	12	1.8	1.0	3.2	57	**10.3**	7.8	13.4
Sjögren syndrome	41	**5.5**	4.0	7.5	28	**5.8**	3.8	8.4	8	**5.0**	2.1	9.8
Systemic lupus erythematosus	28	**6.6**	4.4	9.6	9	**3.0**	1.4	5.7	21	**8.4**	5.2	12.9
Systemic sclerosis	10	**3.0**	1.4	5.5	5	2.2	0.7	5.2	1	0.4	0.0	2.1
Ulcerative colitis	46	**1.7**	1.2	2.3	20	1.2	0.7	1.8	22	1.4	0.9	2.2
Wegener granulomatosis	9	1.5	0.7	2.9	4	1.0	0.3	2.5	12	1.9	1.0	3.4
All	603	**1.6**	1.5	1.8	282	**1.3**	1.1	1.4	371	**2.0**	1.8	2.2

Bold type indicates that the 95% CI does not include 1.00. Abbreviations: O, observed; SIR, standardized incidence ratio; CI, confidence interval.

## Discussion

Our studies on AD and cancer have been very large as they have covered all hospitalized cases of AD in Sweden over an extended period. However, an essential question is whether they find support in other studies and whether they can be generalized. The international InterLymp study reported associations of NHL subtypes with 16 ADs (924 cases) [7]. The study reported 6 associations for DLBCL and 4 of these were significant in the present study; the most common AD, rheumatoid arthritis, showed a risk of 1.94 in that study and 2.16 in our study; a Danish-Swedish case-control study reported a risk of 1.8 [13]. The highest association of follicular lymphoma was with Sjogren syndrome in the InterLymp and our study. The InterLymp study reported no significant associations for CLL, mantle cell lymphoma or ALL in accordance with the few associations in our Table 1 (InterLymp reported a non-significant risk of 2.76 for ALL in type 1 diabetes while our significant risk was 2.75). The previous Nordic (Swedish-Danish cohort) data, summarized by Goldin and Landgren, found a high risk of Hodgkin lymphoma in sarcoidosis patients (14.1, present result 10.3) and in systemic lupus erythematosus patients (5.8, present result 8.4) [6]. These comparisons tend to suggest a relative uniformity of data on B-cell neoplasms in AD patients between large studies.

The InterLymph data as well as ours on B- and T-cell NHL in AD patients showed that both types of cells were vulnerable to transformation by autoimmune stimulation and that there was not much selectivity whether the initial immune activation was through B-cells (e.g., rheumatoid arthritis) or T cells (e.g., celiac disease) [7, 11]. T-cells are nevertheless important in GC processes because they help to initiate the GC reactions for B-cells, and helper T-cells guide the path of B-cells in the zonal maturation process [8]. A pertinent question is if there are common denominators for the 5 ADs (immune thrombocytopenic purpura, polymyositis/dermatomyositis, rheumatoid arthritis, Sjogren syndrome and systemic lupus erythematosus) that jointly associated with GC-derived neoplasms, Firstly, they all present with circulating auto-antibodies and all are systemic ADs, except for immune thrombocytopenic purpura which has thrombocytes as its target [6]. Secondly, these diseases often occur together, including secondary Sjogren syndrome (i.e., presenting with another connective tissue diseases), systemic lupus erythematosus, rheumatoid arthritis and polymyositis/dermatomyositis [14]. It can only be speculated that autoimmune stimulation can critically interfere with the rapid cell division, somatic hypermutation, class switch recombination and immunological selection of maturing B-cell in the GC and delivers damage contributing to transformation [8]. In support, mutation frequencies are known to be one order of magnitude higher in B-cell lymphomas compared to ALL and CLL while the frequency in myeloma is intermediary [15].

In conclusion, we propose here a model, based on the developmental origin of B-cells, explaining why autoimmune stimulation differentially affects B-cell neoplasms and why certain ADs appear to exert selectivity among the target neoplasms and the magnitude of risk. A further mechanistic understanding of the cellular events in B-cell neoplasia will help to validate the model.
